# Implementation and evaluation of a quality and safety tool for ambulatory strongyloidiasis patients at high risk of adverse outcome

**DOI:** 10.1186/s40794-019-0080-1

**Published:** 2019-04-03

**Authors:** Sabrina H. M. Yeung, Omar Mourad, Michael Klowak, Adrienne J. Showler, Stefanie Klowak, Andrea K. Boggild

**Affiliations:** 1 University of Toronto, 27 King’s College Circle, Toronto, Ontario M5S1A1 Canada; 2 Western University, 1151 Richmond Street, London, Ontario N6A 3K7 Canada; 3 McMaster University, 1280 Main Street West, Hamilton, Ontario L8S4L8 Canada; 4 Georgetown University, 3800 Reservoir Rd NW, Washington, DC 20007 USA; 5 Tropical Disease Unit, Toronto General Hospital, 200 Elizabeth Street 13EN-218, Toronto, Ontario M5G2C4 Canada; 6 Public Health Ontario Laboratory, 661 University Avenue, Toronto, Ontario M5G1M1 Canada; 7 Department of Medicine, University of Toronto, Toronto, Canada

**Keywords:** Quality improvement, Patient safety, Strongyloidiasis, Soil-transmitted helminths, Neglected tropical diseases

## Abstract

**Background:**

Strongyloidiasis is a common infection in Canadian migrants that can cause life-threatening hyperinfection in immunosuppressed hosts. We designed and implemented a safety tool to guide management of patients with *Strongyloides* in order to prevent adverse outcomes. Methods: Patients treated at our centre for strongyloidiasis from January 1, 2013 to December 31, 2015 were identified through our ivermectin access log. Patients were categorized into pre-implementation and post-implementation groups. A retrospective chart review for predefined variables was conducted.

**Results:**

Of 37 patients with strongyloidiasis, 26 were in the pre-implementation group and 11 were in the post-implementation group. Documented seroreversion (positive to negative) occurred in 42.1% of patients pre-implementation and 62.5% of patients post-implementation (*p* = 0.420). Documented stool clearance occurred in 80.0% of patients pre-implementation and 100.0% of patients post-implementation (*p* = 1.000). More patients were screened for HTLV-1 coinfection post-implementation (80.0%) versus pre-implementation (30.8%) (*p* = 0.011). Loss to follow-up after treatment occurred in 23.1% of patients pre-implementation and 20.0% of patients post-implementation (*p* = 1.000).

**Conclusions:**

The safety tool may be useful in the treatment of patients with strongyloidiasis to improve documentation of patient outcomes and standardize care. Future research should include a powered prospective study.

**Electronic supplementary material:**

The online version of this article (10.1186/s40794-019-0080-1) contains supplementary material, which is available to authorized users.

## Background

Strongyloidiasis is an infectious disease attributed to *Strongyloides stercoralis*, the intestinal roundworm. The number of people infected is estimated to be 30 to 100 million people worldwide [1], and the disease is widely endemic throughout the tropics, including areas in South America, Africa, Southeast Asia and the Caribbean [2]. In Canada, approximately 9–77% of immigrants and refugees are affected by the disease [3], and travelers to and from endemic areas may also be at risk. It is estimated that at least 2.5 million migrants to Canada have simple intestinal strongyloidiasis [4]. Patients who are infected are frequently asymptomatic or have mild symptoms [5]. Although simple intestinal infections of strongyloidiasis are often manageable on an outpatient basis, the infection may persist for years due to the autoinfective capabilities of *S. stercoralis* [5]. Moreover, when the host is immunocompromised the individual may develop disseminated infection or hyperinfection [1]. These severe cases are often fatal, with a mortality rate approaching 90% [1]. Safety tools, such as surgical checklists, are used in clinical medicine in order to improve quality of care and patient safety [6, 7]. Our group recently published the results of the implementation and evaluation of a safety tool used in the treatment of leprosy at our centre [6]. Given the potential complexity of patients with strongyloidiasis, we developed a safety tool to aid physicians in the management of strongyloidiasis and prevent adverse outcomes. The safety tool is intended for use in patients with laboratory evidence of strongyloidiasis.

Patients with strongyloidiasis seen at our centre are treated with ivermectin, the first-line agent, or albendazole, both of which, until recently, were only available in Canada through application to the Special Access Programme (SAP) of Health Canada. As of September 2018, after completion of this study, ivermectin became licensed and commercially available in Canada, while albendazole remains accessible only via the SAP. In order to gain approval through the SAP, all other available drugs on the market must have been considered first, there must be evidence for the indication, and the patient must have a serious or life-threatening condition [8].

This study aims to evaluate the implementation of a patient safety tool for the treatment of strongyloidiasis at our centre. We hypothesized that standardizing care would improve patient outcomes and screening, and promote continued follow-up with patients.

## Methods

### Pre-implementation safety tool development

A targeted literature search around the standard treatment of strongyloidiasis outside of endemic areas was undertaken in order to define the optimal elements for inclusion in a strongyloidiasis safety tool. A systematic review of randomized controlled trials, meta-analyses, large observational studies, and expert consensus papers on strongyloidiasis management was conducted using MEDLINE from inception to October 2014, with the search terms ‘*Strongyloides*’ or ‘strongyloidiasis’ in combination with ‘quality improvement’, ‘patient safety’, ‘treatment’, ‘management’, ‘adverse outcomes’, or ‘protocols’. The search was restricted to humans and English language literature. Grey literature sources were also consulted to aid in the development of the elements to include in the safety tool. Literature review illuminated the common clinical presentations of strongyloidiasis [1, 2, 5, 9], risk factors for complicated infections [2, 5, 10], diagnosis [5, 9], treatment [5, 10, 11], and follow-up [1, 9, 12]. Based on the literature review, an 8-part prototype tool was developed, which addressed pertinent history, physical examination, and laboratory elements, diagnostic testing, variables that might confound the diagnosis or management (e.g., immunosuppressive medications), treatment, and follow-up. Once completed, the safety tool was piloted with the clinical team in our unit to determine user-friendliness and comprehensiveness. Clinicians and administrators in the unit were provided the tool for the purpose of critical appraisal of content and ease of use. Feedback was incorporated through an iterative process, which led to the finalization of an improved version of the safety tool (Additional file 1). The final safety tool was then implemented in the unit.

### Design and setting

Patients who were treated for strongyloidiasis from January 1, 2013 to December 31, 2015 at our centre were identified through our Special Access Programme (SAP) log. Inclusion criteria were: evidence of strongyloidiasis by positive or indeterminate serological test for antibody, or microscopic detection of *Strongyloides* larvae in a clinical specimen such as stool or sputum. A retrospective chart review was performed, and variables were abstracted with a standardized data collection form [see Additional file 2]. The tool was implemented in June 2015 and patients treated without the tool were categorized into the pre-implementation group, while patients seen in consultation or follow-up with the tool at any point in their treatment were categorized into the post-implementation group. Ethics approval and Institutional Review Board approval were granted by the University Health Network.

### Clinical data

Data including patient characteristics, diagnostic testing, follow-up testing, and screening for hyperinfection or disseminated infection risk factors, as outlined in the Canadian national strongyloidiasis guidelines produced by the Committee to Advise on Tropical Medicine and Travel [4], were de-identified and collected. The diagnostic serological tests used were from Public Health Ontario Laboratory (PHOL) using a Health Canada licensed commercial enzyme linked immunosorbent assay detecting *Strongyloides stercoralis*-specific IgG (SciMedx, NJ) (optical density [OD] of > 0.3 is reactive, 0.2–0.3 is indeterminate, < 0.2 is non-reactive), the Centers for Disease Control and Prevention (CDC) in-house *Strongyloides* assay (OD > 1.7 is positive, < 1.7 is negative), or the National Reference Centre for Parasitology (NRCP) in-house *Strongyloides* assay (OD > 0.3 is reactive, 0.2–0.3 is indeterminate, < 0.2 is non-reactive).

Outcomes assessed included: seroreversion; clearance of larval shedding in stool; screening for risk factors at diagnosis; and loss to follow-up after treatment. All variables of interest were previously defined [see Additional file 3]. Blood eosinophilia was defined as eosinophil count > 0.4 × 10^9^/L. Seroreversion was defined as documentation of positive serological test result at baseline decreasing to indeterminate or negative; or a ≥ 60% decrease in OD from baseline after treatment. Patients who had an indeterminate or negative serological test result at baseline were censored from the seroreversion analysis due to the inability to accurately approximate seroreversion. (One patient in the pre-implementation group was also censored because repeat serological testing was negative before treatment was finished). Stool clearance was defined as documentation of a positive stool test for *S. stercoralis* at baseline, and subsequent clearance of shedding after treatment. Clinical stage of strongyloidiasis was defined as per the CATMAT strongyloidiasis guidelines [4]. Loss to follow-up was defined as the patient failing to return to our centre at least once after prescribed therapy. The tool guides physicians to monitor the patient at follow-up visits 1 and 9 months post-treatment [4]. Furthermore, evaluation of response to treatment is completed through repeat serological testing for seroreversion and is completed 9 to 12 months after treatment [1, 4, 12, 13]. Patients were censored as “not assessible” if their outcome status could not be measured at the time of analysis. Patients who were “not assessible” for an outcome were censored from the analysis.

### Statistical analysis

SPSS Statistics Version 20.0 was used to perform all statistical analyses. All categorical data were compared using the two-sided Fisher’s exact test and all continuous variables were compared with the two-sided *t*-test. An alpha level of 0.05 with power of 80% was set as significant.

## Results

### Patient characteristics

During the study period, 37 patients were treated at our centre for strongyloidiasis. Twenty-six (70%) patients were seen pre-implementation of the tool and 11 (30%) patients were seen post-implementation (Table 1). Median age was 43.5 years (range 16–82 years) in the pre-implementation group and 52 years (range 5–85 years) in the post-implementation group (*p* = 0.316). In the pre-implementation group, 65% (*n* = 17) were male, and in the post-implementation group 64% (*n* = 7) were male (*p* = 1.00). No patients had true disseminated infection (as defined by CATMAT [4] with filariform larvae detectable at distant sites in the context of severe clinical disease), while one patient seen pre-implementation (4%) and three patients seen post-implementation (27%) had mild hyperinfection (as defined by CATMAT). The remaining patients (*n* = 33) had a simple intestinal strongyloidiasis, with no significant differences in clinical status between groups (*p* = 0.07). In 13 patients with eosinophilia (35%), 10 (77%) had documented post-treatment reductions in eosinophil count.

**Table 1:** Baseline patient characteristics and test results

Characteristic	Pre-implementation (*n* = 26)	Post-implementation (*n* = 11)	*p*-value
Median age at first visit, years (range)	43.5 (16–82)	52 (5–85)	0.316
Sex (%)			
Male	17 (65.4)	7 (63.6)	
Female	9 (34.6)	4 (36.4)	1.000
*Strongyloides* serological test (%)			
Positive	20 (76.9)	9 (81.8)	
Indeterminate	5 (19.2)	2 (18.2)	
Negative	1 (3.9)	0 (0.0)	1.000
Stool ova and parasite test (%)			
Larvae detected	5 (19.2)	3 (27.3)	
Negative	16 (61.6)	5 (45.4)	
Not tested	5 (19.2)	3 (27.3)	0.694
Eosinophilia^c^ (%)			
Median eosinophil count (bil/L)	0.20^a^	0.38^b^	0.930
Positive	9 (34.6)	4 (36.4)	
Negative	16 (61.5)	6 (54.5)	
Not tested	1 (3.9)	1 (9.1)	0.857
Type of infection^d^ (%)			
Simple intestinal	25 (96.1)	8 (72.7)	
Hyperinfection	1 (3.9)	3 (27.3)	0.070

^a^One patient with no eosinophil value

^b^Two patients with no eosinophil values

^c^eosinophil count > 0.4 × 10^9^/L

^d^as defined by the CATMAT guidelines [4]

### Pre-implementation and post-implementation outcomes

Thirty-five patients were treated with an ivermectin regimen specific to their clinical disease manifestation, as defined by CATMAT [4]. One patient was initially treated with albendazole, and after potential treatment failure then treated with ivermectin. A patient with hyperinfection was treated with both ivermectin and albendazole. After treatment, six patients in the pre-implementation group (23%), and two patients in the post-implementation group (20%) were lost to follow-up (*p* = 1.000) (Table 2).

**Table 2:** Clinical and parasitological outcomes

	Pre-implementation (*n* = 26)	Post-implementation (*n* = 11)	*p*-value
Seroreversion^c^ (%)			
Yes	8 (42.1)	5 (62.5)	
No	11 (57.9)	3 (37.5)^a^	0.420
Clearance of Stool larval shedding (%)			
Yes	4 (80.0)	3 (100.0)	
No	1 (20.0)	0 (0)	1.000
Loss to follow-up (%)			
Yes	6 (23.1)	2 (20.0)	
No	20 (76.9)	8 (80.0)^b^	1.000

^a^One patient not assessible

^b^One patient not assessible

^c^defined as a two-thirds reduction in baseline serologic optical density

Each patient was tested with a baseline diagnostic serological test for antibody either from PHOL, CDC, or NRCP. In the pre-implementation group, 20 patients (77%) had positive baseline serology, while five patients (19%) had indeterminate serologic testing, and one patient (4%) had a negative serologic result in the context of larval stool shedding. Post-implementation, nine patients (82%) had positive baseline serology, and two patients (18%) had indeterminate serologic testing (Table 1). Documentation of seroreversion occurred in eight patients pre-implementation (42%), and five patients post-implementation (63%) (*p* = 0.420) (Table 2).

At least one stool examination for ova and parasites was performed for 21 patients (81%) in the pre-implementation group, and eight patients (73%) in the post-implementation group (*p* = 0.672). At diagnosis, eight patients (22%) were positive for larval shedding in stool, with five patients (19%) from the pre-implementation group and three (27%) from the post-implementation group (Table 1). Larvae were in the rhabditiform stage for five patients (63%) and the stage was unknown for the remaining three patients (37%). Clearance of larval shedding in stool occurred in four patients pre-implementation (80%) and three patients post-implementation (100%) (*p* = 1.000) (Table 2).

At baseline, all patients were screened for immunosuppressive drug use, presence of neoplasms by review of systems and history of age-appropriate screening, current or upcoming organ transplant, diabetes mellitus, and end stage-renal disease regardless of safety tool implementation status (Table 3). Screening for human T-lymphocytic virus 1 (HTLV-1) co-infection through a serological test occurred in eight patients pre-implementation (31%) and eight patients post-implementation (80%) (*p* = 0.011). Eleven patients in the pre-implementation group (42%), and seven patients in the post-implementation group (70%) received a serological test for HIV co-infection (*p* = 0.264). Peripheral eosinophil count was evaluated in 25 patients pre-implementation (96%) and ten patients post-implementation (90%) (*p* = 0.512). Three patients in the pre-implementation group (12%) and four patients in the post-implementation group (36%) had documentation of evaluation for a potential drug-drug interaction with their current and prescribed therapy (*p* = 0.272).

**Table 3:** Evaluation of risk factors for hyperinfection and disseminated infection

Risk factor screened by specific testing or evaluated based on history and physical examination	Pre-implementation (*n* = 26)	Post-implementation (*n* = 11)	*p*-value^a^
Immunosuppressive drug (%)			
Yes	26 (100.0)	11 (100.0)	
No	0 (0.0)	0 (0.0)	
HTLV-1 ^b^ (%)			
Yes	8 (30.8)	8 (80.0)	
No	18 (69.2)	2 (20.0)	0.011
HIV ^c^ (%)			
Yes	11 (42.3)	7 (70.0)	
No	15 (57.7)	3 (30.0)	0.264
Neoplasms (%)			
Yes	26 (100.0)	11 (100.0)	
No	0 (0.0)	0 (0.0)	–
Upcoming or current organ transplant (%)			
Yes	26 (100.0)	11 (100.0)	
No	0 (0.0)	0 (0.0)	–
Diabetes mellitus (%)			
Yes	26 (100.0)	11 (100.0)	
No	0 (0.0)	0 (0.0)	–
End-stage renal disease (%)			
Yes	26 (100.0)	11 (100.0)	
No	0 (0.0)	0 (0.0)	–
Peripheral or unexplained eosinophilia (%)			
Yes	25 (96.2)	10 (90.9)	
No	1 (3.8)	1 (9.1)	0.512
Drug-drug interaction with prescribed therapy, where patient taking at least 1 other prescription medication (%)			
Yes	3 (11.5)	4 (36.3)	
No	9 (34.6)	2 (18.2)	
Not applicable	14 (53.8)	5 (45.5)	0.272
Country of birth (%)			
Yes	20 (76.9)	11 (100)	
No	6 (23.1)	0 (0)	0.151

^a^p-values are not applicable for results that cannot be compared

^b^One patient not applicable in the post-implementation group

^c^One patient not applicable in the post-implementation group

## Discussion

Our study is the first to document the effects of a safety tool on the standardization of care and outcomes for patients with strongyloidiasis. Patients are intended to return to our centre after treatment to ensure drug therapy adherence, monitoring of symptoms when present, and to perform repeat stool and serologic testing.

We detected no difference in loss to follow-up rates after implementation of the tool, with one in five patients with strongyloidiasis failing to return for care post-treatment across arms. Similarly, seroreversion was documented in 62.5% of patients post-implementation versus 42.1% of patients pre-implementation, which did not differ significantly. Documenting evidence of seroreversion is important given the small risk of treatment failure leading to persistent infection. In a case series, 88 patients with uncomplicated strongyloidiasis were treated with thiabendazole or ivermectin at 200 μg/kg for one or two days [14]. Three months after treatment, there were patients in the thiabendazole and 1-day ivermectin groups that were still positive for the parasite in fecal specimens [14]. Furthermore, in a randomized control trial allocating patients to either treatment with albendazole or ivermectin at 200 μg/kg as a single dose or two doses two weeks apart, there were treatments failures in both groups as demonstrated by persistent larval shedding post-treatment [11]. These studies support that patients who are inadequately treated are at risk of ongoing chronic infection, and, under future immunosuppressive circumstances, may be at risk of adverse outcomes due to hyperinfection and dissemination. The safety tool may therefore help with standardizing care and documentation of seroreversion post-treatment, as it explicitly guides physicians to follow-up with patients at 9-months post-treatment in order to test for seroreversion, and additionally, to create another treatment plan if the treatment had failed. Since the baseline serological test result should also be recorded in the tool, it may also help to ensure assay consistency. For patients shedding larvae in their stool at baseline, it is also important to confirm clearance of shedding post-treatment as a marker of treatment response. Of the patients who were shedding larvae in stool specimens at diagnosis, 80% of patients pre-implementation and 100% of patients post-implementation had documented clearance of shedding of larvae in stool, with no significant difference between groups detected. Potential strategies to improve documentation of seroreversion and stool clearance include educating patients on the importance of repeat serological and stool testing; booking post-treatment serological testing at treatment initiation; and setting patient reminders to return to clinic at the appropriate time point following treatment, though these strategies warrant prospective evaluation to understand their efficacy. Although there were no significant differences in seroreversion and clearance of larval shedding following implementation of the tool, the trends towards higher rates of both in the post-implementation group may suggest improvements in standardization of care. Likewise, in screening for risk factors that could confound the overall management approach to strongyloidaisis, there were post-implementation trends towards increased screening for HIV co-infection, potential drug-drug interactions, and country of birth. Future enrolment of a larger prospective cohort is warranted to best interrogate the tool’s effect on the trends noted in quality outcomes improvement.

We have demonstrated that screening for HTLV-1 coinfection, a major risk factor for *Strongyloides*-associated morbidity and mortality, was greater in the safety tool post-implementation group. HTLV-1 is a retrovirus that persists in hosts for life and is often asymptomatic [15], features which are similar to infections with *Strongyloides*. HTLV-1 has the highest prevalence in Japan, Africa, the Caribbean islands, and South America [16], areas which overlap with *Strongyloides* endemicity, leading to possible co-infection. Patients that are co-infected with HTLV-1 and *Strongyloides* may be at risk of developing a complicated infection [17, 18] and potential treatment failure [19, 20]. In a study of patients with *Strongyloides* hyperinfection, the seroprevalence of HTLV-1 infection was significantly higher in the patients with hyperinfection versus the healthy controls and patients with intestinal strongyloidiasis [18]. Furthermore, a study found that in their cohort of patients with intestinal strongyloidiasis who experienced treatment failure, the seroprevalence of the HTLV-1 was 74.5% [19]. Treatment failure has been suggested to be due to increased expression of IFN-γ and TGF-β1 due to HTLV-1 infection, causing a decrease in IgE and increase in IgG4 [20]. The decrease in IgE may impair mast cell degranulation, and an increase in IgG4 may interfere with IgE activity [20]. Meanwhile, IFN-γ and TGF-β1 have been suggested to decrease IgE production, mast cell activity, and eosinophil activity [20]. These immunological processes may impair the patient’s immune system’s ability to control the *Strongyloides* infection. Therefore, testing for HTLV-1 is imperative in patients with strongyloidiasis, particularly those shedding larvae in the stool, to help predict whether a patient is at risk of developing a complicated infection or treatment failure, so that they can be managed accordingly. Although the post-implementation group had higher rates of screening for HTLV-1 co-infection, it is unknown whether this affected the overall treatment response, as seroreversion and stool clearance rates were similar between the two groups. The number of patients with HTLV-1 co-infection in the study was low, and it is possible that higher numbers of patients detected with a co-infection using the safety tool and subsequently treated for both infections, could affect treatment responses.

The limitations of this study warrant acknowledgment. While implementation of the tool was associated with a significant improvement in screening for HTLV-1 co-infection, it is possible that significant differences in the remaining risk factors between groups could emerge with a larger total sample size. Furthermore, this study occurred at a single specialist centre, where presumably patients were already receiving high-quality strongyloidiasis care during the pre-implementation phase of the study. This is corroborated by our demonstration of patients receiving a diagnostic serological test at baseline and being evaluated for multiple dissemination risk factors pre-implementation. Given the retrospective nature of the analysis and funding limitations, we were unable to test paired baseline (pre-treatment) and post-treatment sera, which necessitated independent diagnostic assays (using the same platform) at separate time points. Given the inherent inter-run variability of EIA-based serologic tests, it is possible that interpretation of post-treatment ODs led to misallocation of some patients into the seroreversion or treatment failure groups. Although paired testing of sera on the same EIA plate is ideal and reduces the likelihood of such misclassifications, our data best reflect the true clinical scenario where patients will be tested using the same diagnostic assay but over time. All of the EIA-based diagnostic assays utilized in this analysis are clinically validated (and in one case, commercial and licensed) reference tests with a long history of performance characteristic stability over time, and have been well published [9, 21–23]. Thus, we believe this potential bias to be small. Another limitation of our study is that there was a greater proportion of patients with hyperinfection seen in the post-implementation phase, though the total number of hyperinfection patients was still very small. It is possible that the observed improvements in documentation of treatment outcomes, safety recommendation evaluation, and follow-up rates were influenced by the severity of the cases seen in the post-implementation phase. Patients with a severe infection may be more likely have risk factors evaluated, adhere to treatment, and to return to the treatment centre after treatment to receive follow-up testing. Furthermore, our results are mainly descriptive due to the small sample size and retrospective nature of the study.

The advantages of using a safety tool include potential improvement in the management of strongyloidiasis through provision of specific and explicit screening, diagnosis, and treatment recommendations, and guidance to document that patients have been adequately treated. A potential weakness is that some physicians may find completing the tool time consuming, although our experience has been that the tool can be used as a form of objective documentation in the patient medical record. However, due to the severity of hyperinfection/disseminated infection, identification of risk factors and the treatment plan may be identified as sections to be prioritized. Given the severity of adverse outcomes that can occur if a strongyloidiasis infection is suboptimally managed, the benefits of a safety tool should theoretically outweigh the potential limitations, though again, this warrants prospective future investigation in a larger cohort.

## Conclusions

In summary, the results of our study suggest that a strongyloidiasis-specific safety tool may be beneficial in standardizing care, and had demonstrable effect on pre-treatment screening for HTLV-1 coinfection, a major risk factor for *Strongyloides* hyperinfection/dissemination, and mortality. Future studies that follow a larger number of strongyloidiasis patients prospectively over a longer period of time to evaluate health and economic outcomes related to safety tool implementation should be pursued.

## Additional files

Additional file 1: Safety tool for the management of ambulatory strongyloidiasis patients. (DOC 250 kb)

Additional file 2: Evaluation of safety tools for ambulatory tropical medicine patients at risk of adverse outcome: data collection form. (PDF 72 kb)

Additional file 3: Patient outcomes and definitions. (PDF 53 kb)
