# Supplementary material for: Implementation and evaluation of a quality and safety tool for ambulatory strongyloidiasis patients at high risk of adverse outcome
Source: Trop Dis Travel Med Vaccines. 2019 Apr 3;5:3. doi: 10.1186/s40794-019-0080-1 (PMC6448213; doi:10.1186/s40794-019-0080-1)
Supplement: Supplementary file 1 — Safety tool for the management of ambulatory strongyloidiasis patients. (DOC 250 kb) [file 40794_2019_80_MOESM1_ESM.doc]

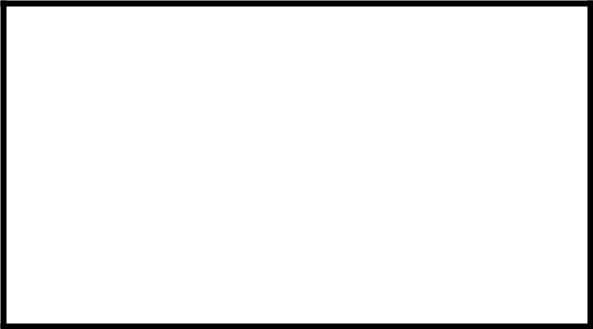
Additional file 1

**Safety Tool for the Management of Ambulatory Strongyloidiasis Patients**

**Physician Name:**

**Date:**

**Part 1 – Patient History**

**
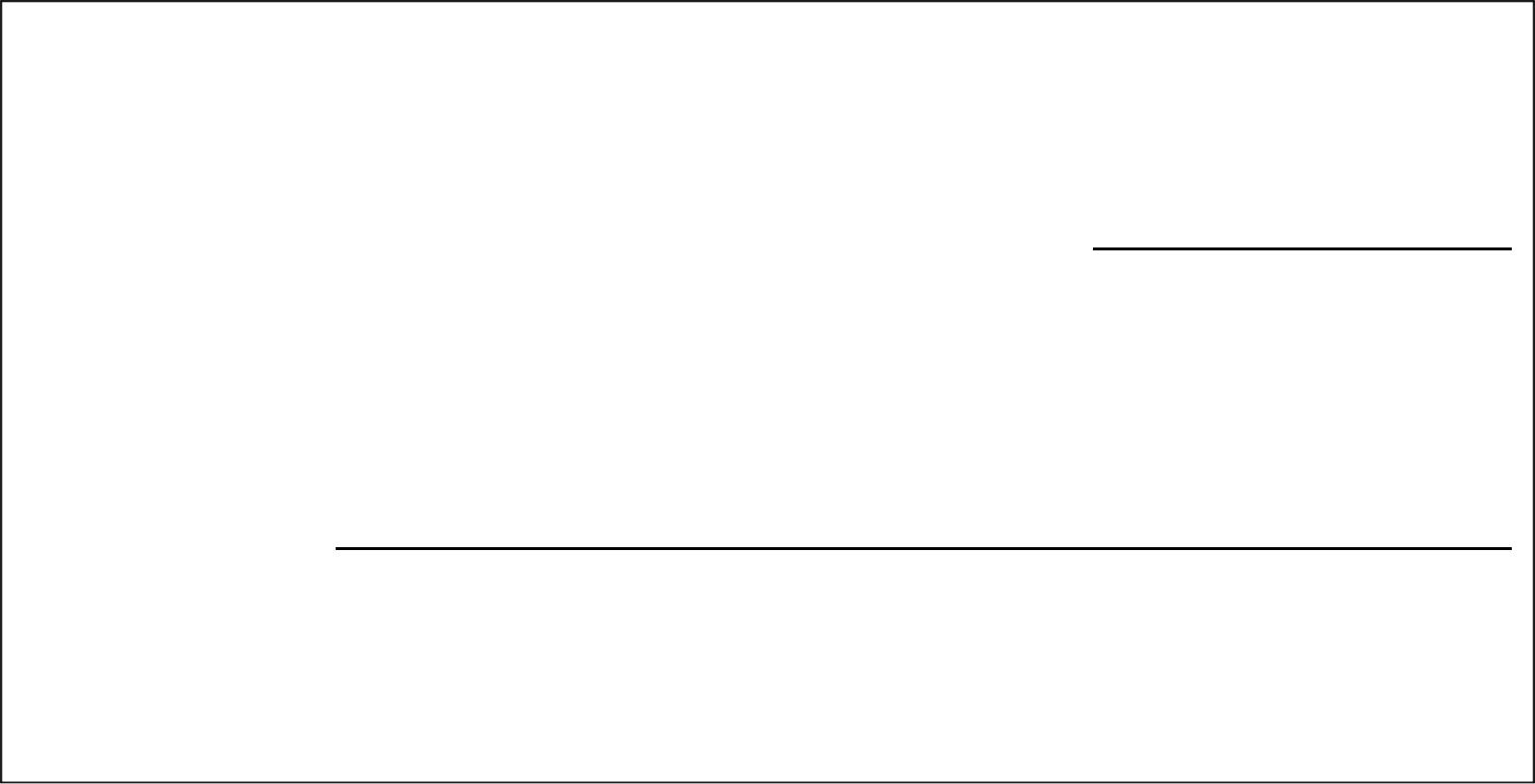
**

_________________________________________________________

_________________________________________________________

_________________________________________________________

_________________________________________

_________________________________________________________

_________________________________________________________

_________________________________________________________

_________________________________________________________

____________

_________________________________________________________

_________________________________________________________

**Part 2 – Patient Diagnosis**

**Serologic result: OD: ____________ Assay: ____________ Date: _____________**

**Stool O&P results (x3) __________________ Date: _____________**

**__________________ Date: _____________**

**__________________ Date: _____________**

**Sputum/Bronchial Wash O&P result: ______________ Date: _____________**

**(Both serologic and stool O&P testing should be ordered for all patients, sputum/bronchial wash testing depends on the clinical syndrome)**

**Patient Signs and Symptoms:**

Does the patient exhibit any symptoms and/or signs that may be suggestive of strongyloidiasis?

HPI:


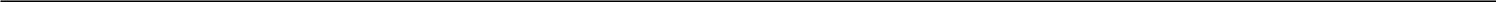

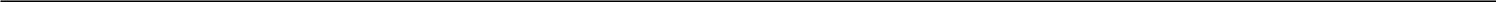

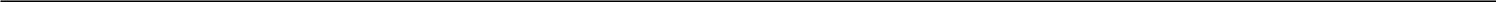


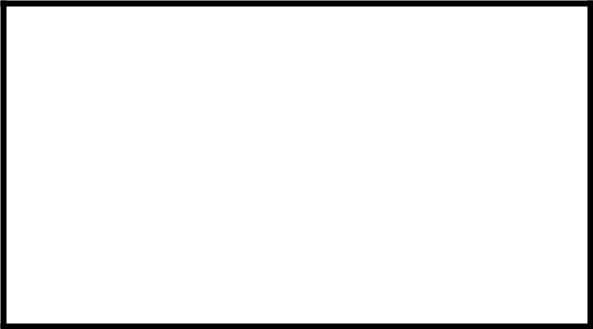
Additional file 1

| **Eosinophilia** | Yes | No | Value: | | | | | Date: ______________ | | |
| --- | --- | --- | --- | --- | --- | --- | --- | --- | --- | --- |
| **Larva Currens** | Yes | No |  |  |  |  |  |  |  |  |
| **Abdominal Pain** | Yes | No | Quality: | |  | | |  |  |  |
|  |  |  | Severity: | | |  | |  |  |  |
|  |  |  | Frequency: | | | |  |  |  |  |
| **Diarrhea** | Yes | No | Quality: | |  | | |  |  |  |
|  |  |  | Severity: | | |  | |  |  |  |
|  |  |  | Frequency: | | | |  |  |  |  |
| **Weight Loss:** | Yes | No | Severity: | | | | |  |  |  |
|  |  |  |  |  |  |  |  |  |  |  |


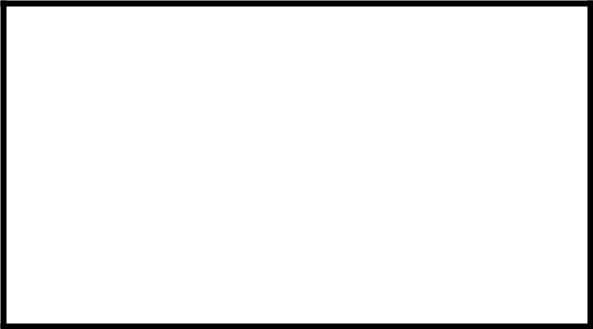
Additional file 1

**Part 3 – Points of Concern**

The following section will look at specific screening questions that will aid in the management and prevention of severe sequelae of strongyloidiasis including disseminated infection and hyperinfection.

| Is the patient currently on or about to be put on | **Yes** | | **No** | |
| --- | --- | --- | --- | --- |
| immunosuppressive drugs? | **Comments:** | |  |  |
| Has the patient's serum samples shown the | **Yes** | | **No** | |
| presence of antibodies to HTLV-1 | **Comments:** | |  |  |
| Has the patient’s serum shown the presence of | **Yes** | | **No** | |
| HIV antibodies? | **Comments:** | |  |  |
| Does the patient have any neoplasms, | **Yes** | | **No** | |
| particularly hematologic malignancies such as | **Comments:** | |  |  |
| lymphoma or leukemia? |  |  |  |  |
| Has the patient undergone, or are they being | **Yes** | | **No** | |
| evaluated to undergo, an organ transplant? | **Comments:** | |  |  |
|  |  |  |  |  |
| Does the patient have diabetes mellitus? | **Yes** | | **No** | |
|  | **Comments:** | |  |  |
| Does the patient suffer from end-stage renal | **Yes** | | **No** | |
| disease? | **Comments:** | |  |  |
|  |  |  |  |  |
| Does the patient suffer from any rheumatic | **Yes** | | **No** | |
|  | **Comments:** | | **_** | |
| diseases such as rheumatoid arthritis? |  |  |  |  |
|  | **Yes** |  | **No** |  |
| Does the patient have any malabsorption issues |  |  |  |  |
|  | **Comments:** | |  |  |
| or are they currently suffering from |  |  |  |  |
|  |  |  |  |  |
| malnutrition? |  |  |  |  |

Does the patient have peripheral or unexplained eosinophilia?

Patient’s country of birth

**Current Medications:**

**Yes** **No**

**Comments:**

**
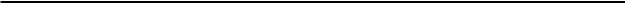

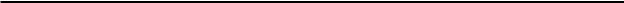
**

**If you answered yes to any of these (or the patient is from an endemic country) questions, immediate screening by stool and sputum O&P examination is necessary as these patients are at high risk of disseminated strongyloidiasis or hyperinfection syndrome.**


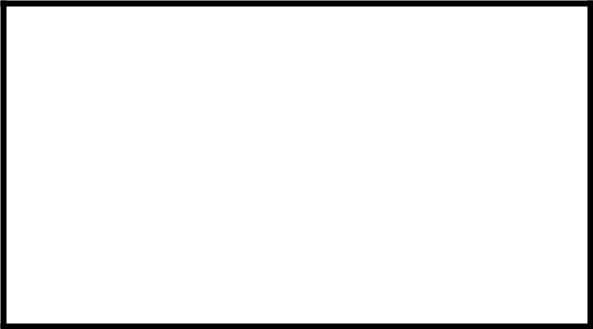
Additional file 1

**Part 4 – Physical Examination**

**Vitals:** Temp: HR: _________ BP: RR: O2 sat: _________

**Head & Neck:**

**CVS:**

**Resp:**

**Abdo:**

**Skin:**

**MSK:**

**Neuro:**

**Part 5 – Assessment**

**
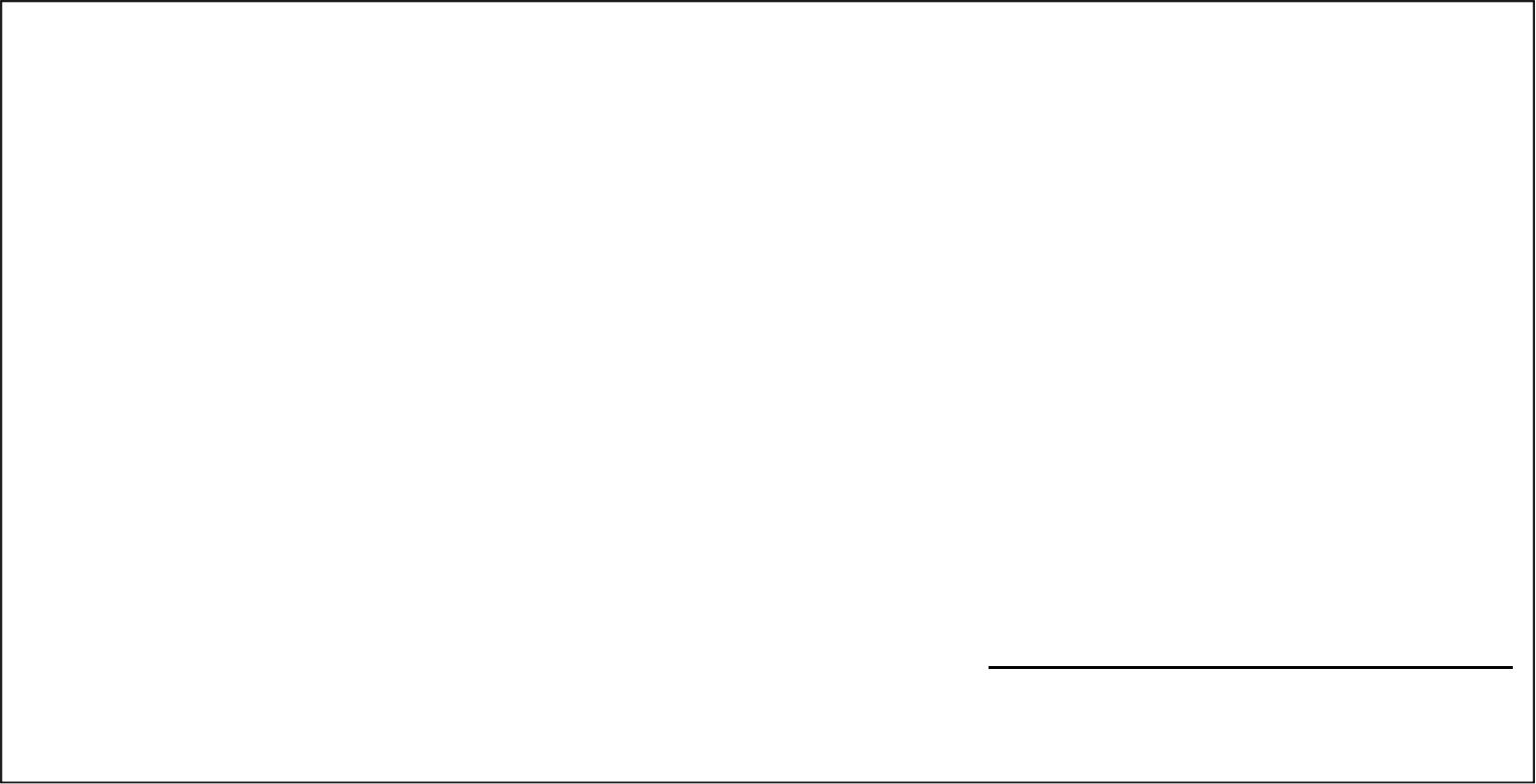
**

_________________________________________________________

_________________________________________________________

_________________________________________________________

_________________________________________________________

_________________________________________________________

_________________________________________________________

_________________________________________________________

_________________________________________________________

_________________________________________________________

_________________________________________________________

_____________________________________


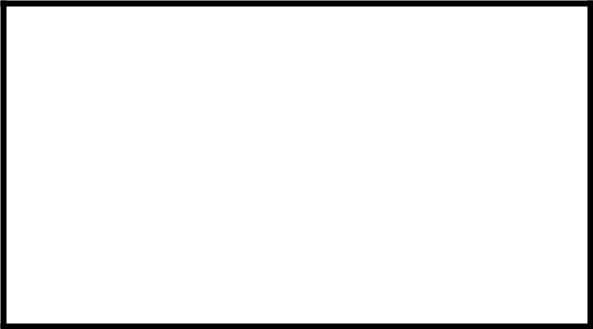
Additional file 1

**Part 6 – Treatment**

**First Line therapy:** Ivermectin – SINGLE DOSE p.o, 200 µg/kg x 2 DAYS, 14-days apart

| **Alternative:** Albendazole – 400 mg orally TWICE per day for 7 DAYS |  |  |
| --- | --- | --- |
| **Part 6A** |  |  |
| Does the patient have a confirmed or suspected Loa Loa infection? | **Yes** | **No** |
| Is the patient pregnant or lactating? | **Yes** | **No** |
| Does the patient weigh less than 15 kg? | **Yes** | **No** |

**Part 6B**

Is the patient hypersensitive to benzimidazoles? **Yes** **No**

**If you selected NO for all questions in section 5A prescribe IVERMECTIN. If you selected YES for any questions in section 5A prescribe ALBENDAZOLE. If you answered YES for questions in both 5A and 5B then further considerations are necessary.**

**Part 6C – Proposed Drug Regimen**

**Ivermectin or Albendazole:**

**Part 7 – Plan**

| **Completion of SAP Forms for drug of choice:** | | **Yes** | **No** | **N/A** | **Notes:** | | |
| --- | --- | --- | --- | --- | --- | --- | --- |
| **Counselling of patient regarding:** | |  |  |  |  |  |  |
|  |  | **Yes** | **No** | **N/A** | **Notes:** |  | |
| Drug-drug interactions: | |  |  |  |  |  |  |
| Medication Side Effects: | | **Yes** | **No** | **N/A** | **Notes:** |  | |
| Dosing and administration: | | **Yes** | **No** | **N/A** | **Notes:** |  | |
| Handout given: | | **Yes** | **No** | **N/A** | **Notes:** | | |
| **Medication** | |  |  |  |  |  |  |
| Dispensed at visit: | | **Yes** | **No** | **N/A** | **Notes:** |  | |
| For pick-up: | | **Yes** | **No** | **N/A** | **Notes:** |  | |
| To be mailed: | | **Yes** | **No** | **N/A** | **Notes:** | | |
| **Bloodwork:** |  |  |  |  |  |  |  |

**Follow-up Appt: _______________________________________________________________________**


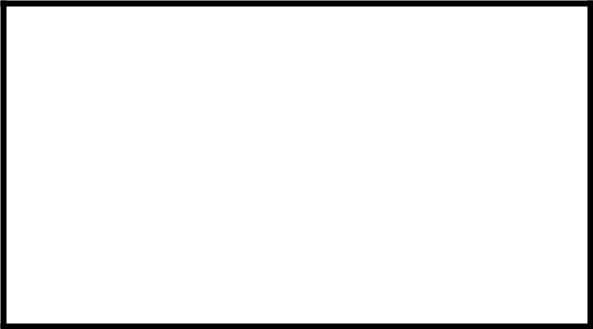
Additional file 1

**Part 8A – 1 month Follow Up**

**Physician Name:**

**Date:**

**Acute and Chronic Strongyloidiasis**

Follow up should be done 1- and 9-months post-treatment

| Do the repeat stool samples (x3) show signs of larval shedding? If so, list stage. | **Yes** | **No** | **Stage: ______________** | |
| --- | --- | --- | --- | --- |
|  |  |  |  |  |
| Has the patient tolerated the drug? | **Yes** | **No** | **Notes:** | |
|  |  |  |  |

**Ongoing shedding of larvae in the stool indicates treatment failure.**

**If the patient has failed initial therapy, please describe the plan:**

**
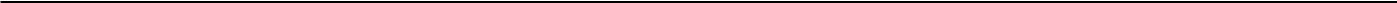

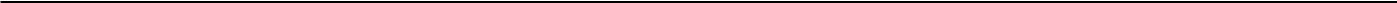

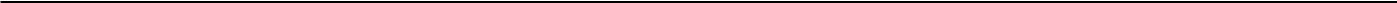

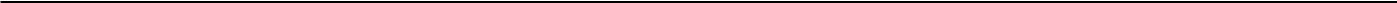
**

| Patient will be prescribed another course of | **Yes** | **No** |
| --- | --- | --- |
| Ivermectin: |  |  |

Dose and duration: _____________________________________________________________________

**After treatment has been administered, follow up again after 1- and 9-months for serological and/or stool sample testing as appropriate. Please fill out Part 8B.**

**Part 8B**

| **Completion of SAP Forms for drug of choice:** | | | **Yes** | **No** | **N/A** | **Notes:** | | |
| --- | --- | --- | --- | --- | --- | --- | --- | --- |
| **Counselling of patient regarding:** | | |  |  |  |  |  |  |
| Drug-drug interactions: | | | **Yes** | **No** | **N/A** | **Notes:_______________** | | |
| Medication Side Effects: | | | **Yes** | **No** | **N/A** | **Notes:** | | |
|  |  |  |  |  |  |  |  |  |
| Dosing and administration: | | | **Yes** | **No** | **N/A** | **Notes:** | | |
|  |  |  |  |  |  |  |  |  |
| Handout given: | | | **Yes** | **No** | **N/A** | **Notes:** | | |
| **Medication** | | |  |  |  |  |  |  |
| Dispensed at visit: | | | **Yes** | **No** | **N/A** | **Notes:** | | |
|  |  |  |  |  |  |  |  |  |
| For pick-up: | | | **Yes** | **No** | **N/A** | **Notes:** | | |
|  |  |  |  |  |  |  |  |  |
| To be mailed: | | | **Yes** | **No** | **N/A** | **Notes:** | | |
| **Bloodwork:** |  | |  |  |  |  |  |  |
| **Follow-up Appt: ________________________** | |  |  |  |  |  |  |  |


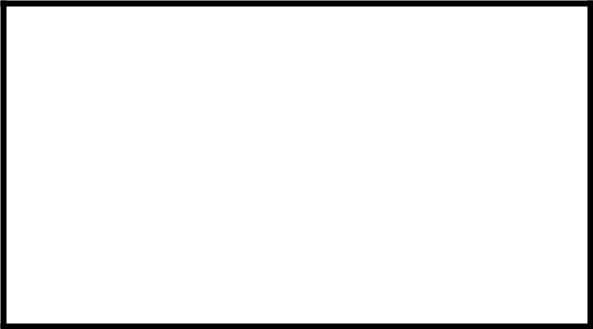
Additional file 1

**Part 8C – 9 month Follow Up**

**Physician Name:**

**Date:**

Follow up should be done 1- and 9-months post-treatment

| Does repeat serology demonstrate antibodies | **Yes** | **No** | **OD:** |
| --- | --- | --- | --- |
| to Strongyloides? If so, note optical density. | **Yes** | **No** |  |
| If repeat serology is positive, has there been a |  |
|  |  |  |
| two-thirds reduction in antibody OD compared |  |  |  |
| to initial OD? |  |  |  |

**Failure to achieve a two-thirds reduction in OD 9- to 12-months following treatment may indicate failure.**

**If the patient has failed initial therapy, please describe the plan:**

**
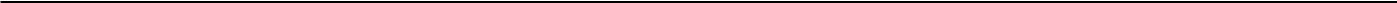

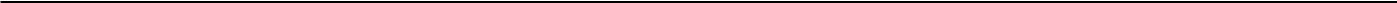

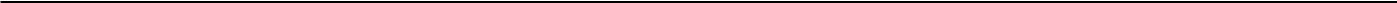

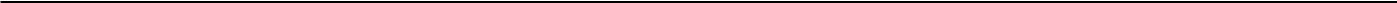
**

Patient will be prescribed another course of Ivermectin: **Yes** **No**

Dose and duration: _____________________________________________________________________

**After treatment has been administered, follow up again after 1- and 9-months for serological and/or stool sample testing as appropriate. Please fill out Part 8D on Page 8.**

**Part 8D**

| **Completion of SAP Forms for drug of choice:** | | **Yes** | **No** | **N/A** | **Notes:** | |  |
| --- | --- | --- | --- | --- | --- | --- | --- |
| **Counselling of patient regarding:** | |  |  |  |  |  |  |
| Drug-drug interactions: | | **Yes** | **No** | **N/A** | **Notes:** | |  |
|  | |  |  |  |  |  |  |
| Medication Side Effects: | | **Yes** | **No** | **N/A** | **Notes:** | |  |
|  | |  |  |  |  |  |  |
| Dosing and administration: | | **Yes** | **No** | **N/A** | **Notes:** | |  |
|  | |  |  |  |  |  |  |
| Handout given: | | **Yes** | **No** | **N/A** | **Notes:** | |  |
| **Medication** | |  |  |  |  |  |  |
| Dispensed at visit: | | **Yes** | **No** | **N/A** | **Notes:** | |  |
|  | |  |  |  |  |  |  |
| For pick-up: | | **Yes** | **No** | **N/A** | **Notes:** | |  |
|  | |  |  |  |  |  |  |
| To be mailed: | | **Yes** | **No** | **N/A** | **Notes:** | |  |
| **Bloodwork:** |  |  |  |  |  |  |  |

**Follow-up Appt: _______________________________________**
