# Supplementary material for: Implementation and evaluation of a quality and safety tool for ambulatory strongyloidiasis patients at high risk of adverse outcome
Source: Trop Dis Travel Med Vaccines. 2019 Apr 3;5:3. doi: 10.1186/s40794-019-0080-1 (PMC6448213; doi:10.1186/s40794-019-0080-1)
Supplement: Supplementary file 2 — Evaluation of safety tools for ambulatory tropical medicine patients at risk of adverse outcome: data collection form. (PDF 72 kb) [file 40794_2019_80_MOESM2_ESM.pdf]

Additional file 2

Evaluation of Safety Tools for Ambulatory Tropical Medicine Patients at Risk of Adverse Outcome: Data Collection Form

Safety Tool: Strongyloidiasis Case #: ______________________________

Pre or Post Safety Tool Implementation: ____

Type of Strongyloidiasis: Simple Hyperinfection Disseminated

General Information

Sex: M F

Age at first visit: _________ Date of first visit: ___________

Symptoms Documented: Y/ N Physical Exam: Y/ N

Safety recommendations

Patient was evaluated for immunosuppressive drugs Y / N/ U / NA

Patient was evaluated for HTLV-1 Y / N / U / NA

Patient was evaluated for HIV Y / N / U / NA

Patient was evaluated for neoplasms Y / N / U / NA

Patient was evaluated for upcoming or current organ transplant Y / N / U / NA

Patient was evaluated for diabetes mellitus Y / N / U / NA

Patient was evaluated for end-stage renal disease Y / N / U / NA

Patient was evaluated for peripheral or unexplained eosinophilia Y / N / U / NA

Patient's country of birth _______________________________

Patient was evaluated for DDI Y / N / U / NA

Treatment

Drug regiment prescribed: Ivermectin /Albendazole

Dose of selected drug: ________________________________

Date taken: ________________________________

Additional file 2

General Labs at Diagnosis

Serology documented: Y/ N / U / NA

Serological results:

OD: ____________ Assay: _____________ Date: _____________

Stool O & P Documented: Y/ N / U / NA

Is the patient shedding larvae?
1. Y / N / U / NA Date: ______________
2. Y / N / U / NA Date: ______________
3. Y / N / U / NA Date: ______________
4. Y / N / U / NA Date: ______________

If Yes the Stage of Larvae: Rhabditiform / Filariform / U / NA

Sputum and Bronchial Wash Documented: Y / N / U / NA

Is the patient shedding larvae?
1. Y/ N/ U / NA Date: ______________
2. Y/ N/ U / NA Date: ______________

If Yes the Stage of Larvae: Rhabditiform / Filariform / U / NA

Eosinophilia test documented: Y / N / U / NA

Eosinophilia presence: Y / N / U / NA Value: ____________ Date: _____________

Y / N / U / NA Value: ____________ Date: _____________

Patient Follow-Up

Follow-Up Month: Date: _____________

Patient Reported Therapy Compliance: Y / N / U / NA

Stool O&P Documented Y/ N / U / NA

Is the patient shedding larvae
1. Y/ N / U / NA Date: ______________
2. Y/ N / U / NA Date: ______________
3. Y/ N / U / NA Date: ______________
4. Y/ N / U / NA Date: ______________

If Yes the Stage of Larvae: Rhabditiform / Filariform / U / NA

Eosinophilia test documented: Y / N / U / NA

Eosinophilia presence: Y / N / U / NA Value: ____________ Date: _____________

Y / N / U / NA Value: ____________ Date: _____________

Serology documented: Y / N / U / NA

Serology (if necessary) OD: ____________ Assay: _____________ Date: _____________

Additional file 2

Patient Follow-Up

Follow-Up Month: Date: _____________

Patient Reported Therapy Compliance: Y / N / U / NA

Stool O&P Documented Y/ N/ U / NA
Is the patient shedding larvae 1. Y/ N/ U / NA Date: ______________
2. Y/ N/ U / NA Date: ______________
3. Y/ N/ U / NA Date: ______________
4. Y/ N/ U / NA Date: ______________

If Yes the Stage of Larvae: Rhabditiform/Filariform/ U / NA
Eosinophilia test documented: Y / N / U / NA
Eosinophilia presence Y / N / U / NA Value: ____________ Date: _____________
Y / N / U / NA Value: ____________ Date: _____________

Serology documented: Y/ N/ U / NA

Serology (if necessary) OD: ____________ Assay: _____________ Date: _____________

Patient Follow-Up

Follow-Up Month: Date: _____________

Patient Reported Therapy Compliance: Y / N / U / NA

Stool O&P Documented Y / N / U / NA
Is the patient shedding larvae 1. Y/ N/ U / NA Date: ______________
2. Y/ N/ U / NA Date: ______________
3. Y/ N/ U / NA Date: ______________
4. Y/ N/ U / NA Date: ______________

If Yes the Stage of Larvae: Rhabditiform/Filariform/ U / NA

Eosinophilia test documented: Y / N / U / NA
Eosinophilia presence Y / N / U / NA Value: ____________ Date: _____________
Y / N / U / NA Value: ____________ Date: ____________

Serology documented: Y/ N/ U / NA

Serology (if necessary) OD: ____________ Assay: _____________ Date: _____________
