# Supplementary material for: Implementation and evaluation of a quality and safety tool for ambulatory strongyloidiasis patients at high risk of adverse outcome
Source: Trop Dis Travel Med Vaccines. 2019 Apr 3;5:3. doi: 10.1186/s40794-019-0080-1 (PMC6448213; doi:10.1186/s40794-019-0080-1)
Supplement: Supplementary file 3 — Patient outcomes and definitions. (PDF 53 kb) [file 40794_2019_80_MOESM3_ESM.pdf]

Additional file 3

Patient outcomes and definitions.

Variable	Definition
Patient outcomes	
Seroreversion	Yes = documentation of follow-up serology test after treatment that demonstrated a negative/indeterminate result or ≥60% decrease from baseline if baseline positive; No = no documentation of follow-up serology test demonstrating seroreversion
Stool larvae shedding clearance	Yes = documentation of follow-up stool ova and parasite testing demonstrating clearance of shedding if baseline positive; No = no documented stool test demonstrating clearance
Loss to follow-up	Yes = patient did not return to clinic after prescribed therapy; No = patient returned to clinic at least once after prescribed therapy
Safety recommendations	
Immunosuppressive drug evaluation	Yes = current medications were evaluated; No = current medication were not evaluated

Additional file 3

HTLV-1 evaluation	Yes = HTLV-1 antibody test was performed or patient has known infection; No = HTLV-1 antibody test was not performed; Not applicable = risk of HTLV-1 infection is very low
HIV evaluation	Yes = HIV antibody and/or antigen test was performed or patient has known infection; No = HIV antibody and/or antigen test was not performed; Not applicable = risk of HIV infection is very low
Neoplasms evaluation	Yes = neoplasms or past medical history was evaluated; No = neoplasms or past medical history was not evaluated
Upcoming or current organ transplant evaluation	Yes = organ transplant or past medical history was evaluated; No = organ transplant or past medical history was not evaluated
Diabetes mellitus evaluation	Yes = diabetes mellitus or past medical history was evaluated; No = diabetes mellitus or past medical history was not evaluated

Additional file 3

End-stage renal disease evaluation	Yes = end-stage renal disease or past medical history was evaluated; No = end-stage renal disease or past medical history was not evaluated
Peripheral or unexplained eosinophilia evaluation	Yes = eosinophil count was measured in the past year; No = eosinophil count was not measured in the past year
Drug-drug interaction with prescribed therapy evaluation	Yes = documentation of evaluation of potential interaction of prescribed therapy with current prescription medications; No = no documentation of evaluation of potential drug-drug interaction; Not applicable = not on current prescription medications or had previously taken prescribed therapy
Country of birth evaluation	Yes = country of birth was documented; No = country of birth was not documented
Eosinophilia	Yes = baseline eosinophils > $0.4x10^9$/L at the closest measurement before treatment or as eosinophilia identified by the treating physician;

Additional file 3

	No = baseline eosinophils ≤ $0.4x10^9/L$ at the closest measurement before treatment and no eosinophilia identified by the treating physician
